# 3-Carb­oxy-5-(pyridinium-4-yl)benzoate: a redetermination

**DOI:** 10.1107/S1600536811016394

**Published:** 2011-05-07

**Authors:** Shi-Jie Li, Dong-Liang Miao, Wen-Dong Song, Shao-Wei Tong

**Affiliations:** a College of Food Science and Technology, Guangdong Ocean University, Zhanjiang 524088, People’s Republic of China; b College of Science, Guangdong Ocean University, Zhanjiang 524088, People’s Republic of China

## Abstract

The title compound, C_13_H_9_NO_4_, crystallizes in a zwitterionic form with the pyridine N atom protonated and the carboxyl OH group deprotonated. The benzene and pyridinium rings are inclined with a dihedral angle of 31.42 (14)° between them. A previous report of this stucture claims, we believe incorrectly, that neither of the carboxyl­ate groups is deprotonated [Zhang *et al.* (2010 ▶). *Acta Cryst.* E**66**, o2928–o2928]. In the crystal, inter­molecular O—H⋯O, N—H⋯O and weak C—H⋯O hydrogen-bonding inter­actions link adjacent mol­ecules into a three-dimensional supra­molecular network.

## Related literature

For coordination polymers based on pyridine­carboxyl­ate ligands, see: Lu & Luck (2003 ▶); Ma *et al.* (2009 ▶). For a previous report of the structure of this mol­ecule, which claims that neither of the carboxyl­ate groups is deprotonated, see: Zhang *et al.* (2010 ▶).
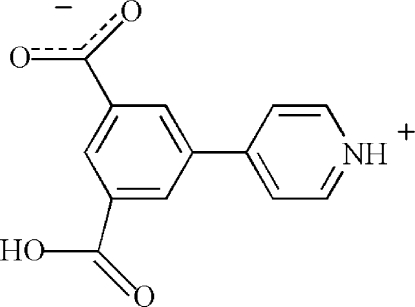


## Experimental

### 

#### Crystal data

C_13_H_9_NO_4_
*M* *_r_* = 243.21
Orthorhombic, 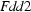

*a* = 15.5702 (13) Å
*b* = 37.377 (3) Å
*c* = 7.2016 (9) Å
*V* = 4191.1 (7) Å^3^
*Z* = 16
Mo *K*α radiation
μ = 0.12 mm^−1^
*T* = 298 K
0.38 × 0.15 × 0.07 mm

#### Data collection

Bruker SMART 1000 CCD area-detector diffractometer
Absorption correction: multi-scan (*SADABS*; Bruker, 2007 ▶) *T*_min_ = 0.957, *T*_max_ = 0.992
5456 measured reflections
1024 independent reflections
885 reflections with *I* > 2σ(*I*)
*R*_int_ = 0.048

#### Refinement

*R*[*F* ^2^ > 2σ(*F* ^2^)] = 0.050
*wR*(*F* ^2^) = 0.142
*S* = 1.09
1024 reflections
164 parameters
4 restraints
H-atom parameters constrained
Δρ_max_ = 0.21 e Å^−3^
Δρ_min_ = −0.26 e Å^−3^

### 

Data collection: *SMART* (Bruker, 2007 ▶); cell refinement: *SAINT* (Bruker, 2007 ▶); data reduction: *SAINT*; program(s) used to solve structure: *SHELXS97* (Sheldrick, 2008 ▶); program(s) used to refine structure: *SHELXL97* (Sheldrick, 2008 ▶); molecular graphics: *SHELXTL* (Sheldrick, 2008 ▶); software used to prepare material for publication: *SHELXTL*.

## Supplementary Material

Crystal structure: contains datablocks global, I. DOI: 10.1107/S1600536811016394/sj5133sup1.cif

Structure factors: contains datablocks I. DOI: 10.1107/S1600536811016394/sj5133Isup2.hkl

Supplementary material file. DOI: 10.1107/S1600536811016394/sj5133Isup3.cml

Additional supplementary materials:  crystallographic information; 3D view; checkCIF report

## Figures and Tables

**Table 1:** Hydrogen-bond geometry (Å, °)

*D*—H⋯*A*	*D*—H	H⋯*A*	*D*⋯*A*	*D*—H⋯*A*
N1—H1⋯O4^i^	0.86	1.70	2.562 (4)	175
N1—H1⋯O3^i^	0.86	2.67	3.252 (4)	126
O1—H1*A*⋯O4^ii^	0.82	1.96	2.643 (5)	141
C8—H8⋯O2^iii^	0.93	2.71	3.632 (5)	171
C10—H10⋯O2^iii^	0.93	2.58	3.225 (6)	127
C9—H9⋯O1^iv^	0.93	2.59	3.316 (6)	135

Symmetry codes: (i) 
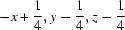
; (ii) 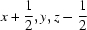; (iii) 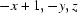; (iv) 
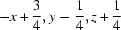
.

**Fig. 1.**
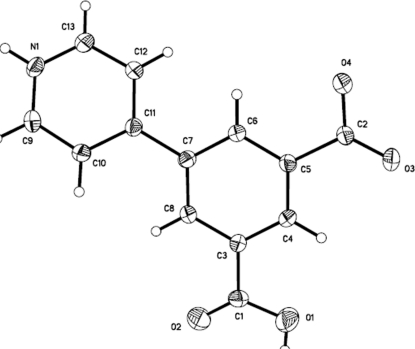
The structure of the title compound, showing the atomic numbering scheme. Non-H atoms are shown with 30% probability displacement ellipsoids.

**Fig. 2.**
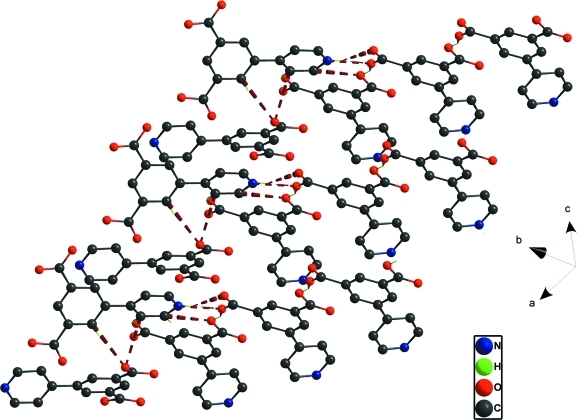
Crystal packing of the title compound (H atoms not involved in forming hydrogen bonds are omitted for clarity).

## supplementary crystallographic information

### Comment

Rigid pyridinecarboxylate ligands have been used extensively to react with metal ions and generate coordination polymers with fascinating structures and properties (Lu & Luck 2003; Ma *et al.*, 2009). As part of an ongoing investigation into coordination polymers based on pyridinecarboxylate ligands, we report here the crystal structure of the title compound.

As shown in Fig. 1, the title compound, C_13_H_9_NO_4_, crystallizes in a zwitterionic form with the pyridine N protonated and one of the carboxyl OH groups deprotonated. The locations of the N and O bound H atoms are clearly shown in a difference Fourier map. A previous report of the same structure in the same space group and with similar unit-cell parameters claims that neither of the carboxylate groups are deprotonated (Zhang *et al.*, 2010). We believe this assignment to be in error. A conformational feature of the molecule is a rigid structure with the benzene and pyridinium rings inclined at an angle of 31.42 (14) ° to one another. In the crystal structure, molecules are interconnected by O—H···O, N—H···O and weak C—H···O hydrogen bonding interactions (Table. 1), generating a three-dimensional supramolecular network (Fig. 2).

### Experimental

Commercially available 5-(pyridin-4-yl)isophthalic acid was further purified by repeated recrystallization from anhydrous ethanol. Colorless crystals suitable for X-ray analysis were obtained by slow evaporation of the ethanol solvent at room temperature.

### Refinement

All H-atoms were positioned geometrically and refined using a riding model with d(C—H) = 0.93 Å, *U*_iso_=1.2*U*_eq_ (C) for aromatic hydrogen atoms 0.86 Å, *U*_iso_ = 1.2*U*_eq_ (N) for the NH group and 0.82 Å, *U*_iso_ = 1.5*U*_eq_ (O) for the OH group. In the absence of significant anomalous dispersion effects, Friedel pairs were merged.

### Crystal data

**Table:**

C_13_H_9_NO_4_	*F*(000) = 2016
*M**_r_* = 243.21	*D*_x_ = 1.542 Mg m^−^^3^
Orthorhombic, *F**d**d*2	Mo *K*α radiation, λ = 0.71073 Å
Hall symbol: F 2 -2d	Cell parameters from 1702 reflections
*a* = 15.5702 (13) Å	θ = 2.5–25.9°
*b* = 37.377 (3) Å	µ = 0.12 mm^−^^1^
*c* = 7.2016 (9) Å	*T* = 298 K
*V* = 4191.1 (7) Å^3^	Block, colorless
*Z* = 16	0.38 × 0.15 × 0.07 mm

### Data collection

**Table:**

Bruker SMART 1000 CCD area-detector diffractometer	1024 independent reflections
Radiation source: fine-focus sealed tube	885 reflections with *I* > 2σ(*I*)
graphite	*R*_int_ = 0.048
φ and ω scans	θ_max_ = 25.2°, θ_min_ = 2.8°
Absorption correction: multi-scan (*SADABS*; Bruker, 2007)	*h* = −15→18
*T*_min_ = 0.957, *T*_max_ = 0.992	*k* = −42→44
5456 measured reflections	*l* = −8→8

### Refinement

**Table:**

Refinement on *F*^2^	Primary atom site location: structure-invariant direct methods
Least-squares matrix: full	Secondary atom site location: difference Fourier map
*R*[*F*^2^ > 2σ(*F*^2^)] = 0.050	Hydrogen site location: inferred from neighbouring sites
*wR*(*F*^2^) = 0.142	H-atom parameters constrained
*S* = 1.09	*w* = 1/[σ^2^(*F*_o_^2^) + (0.0985*P*)^2^ + 0.9031*P*] where *P* = (*F*_o_^2^ + 2*F*_c_^2^)/3
1024 reflections	(Δ/σ)_max_ < 0.001
164 parameters	Δρ_max_ = 0.21 e Å^−^^3^
4 restraints	Δρ_min_ = −0.26 e Å^−^^3^

### Special details

**Table:**

Geometry. All e.s.d.'s (except the e.s.d. in the dihedral angle between two l.s. planes) are estimated using the full covariance matrix. The cell e.s.d.'s are taken into account individually in the estimation of e.s.d.'s in distances, angles and torsion angles; correlations between e.s.d.'s in cell parameters are only used when they are defined by crystal symmetry. An approximate (isotropic) treatment of cell e.s.d.'s is used for estimating e.s.d.'s involving l.s. planes.
Refinement. Refinement of *F*^2^ against ALL reflections. The weighted *R*-factor *wR* and goodness of fit *S* are based on *F*^2^, conventional *R*-factors *R* are based on *F*, with *F* set to zero for negative *F*^2^. The threshold expression of *F*^2^ > σ(*F*^2^) is used only for calculating *R*-factors(gt) *etc*. and is not relevant to the choice of reflections for refinement. *R*-factors based on *F*^2^ are statistically about twice as large as those based on *F*, and *R*- factors based on ALL data will be even larger.

### Fractional atomic coordinates and isotropic or equivalent isotropic displacement parameters (Å^2^)

**Table:**

	*x*	*y*	*z*	*U*_iso_*/*U*_eq_	
N1	0.1480 (2)	−0.05751 (9)	0.2133 (5)	0.0409 (9)	
H1	0.1190	−0.0771	0.2101	0.049*	
O1	0.5435 (2)	0.10547 (10)	0.0930 (7)	0.0710 (12)	
H1A	0.5939	0.1044	0.0606	0.106*	
O2	0.5453 (2)	0.04721 (10)	0.0306 (6)	0.0697 (13)	
O3	0.29114 (19)	0.16581 (7)	0.3311 (6)	0.0532 (9)	
O4	0.18242 (19)	0.13290 (7)	0.4374 (6)	0.0482 (9)	
C1	0.5099 (3)	0.07316 (11)	0.0923 (7)	0.0397 (9)	
C2	0.2558 (2)	0.13670 (10)	0.3568 (7)	0.0360 (9)	
C3	0.4199 (2)	0.07241 (9)	0.1660 (6)	0.0327 (9)	
C4	0.3802 (2)	0.10341 (10)	0.2268 (6)	0.0328 (9)	
H4A	0.4102	0.1249	0.2255	0.039*	
C5	0.2964 (2)	0.10264 (10)	0.2892 (6)	0.0314 (9)	
C6	0.2511 (2)	0.07076 (10)	0.2856 (6)	0.0314 (9)	
H6	0.1940	0.0704	0.3236	0.038*	
C7	0.2898 (2)	0.03945 (10)	0.2260 (6)	0.0315 (9)	
C8	0.3750 (2)	0.04014 (10)	0.1687 (6)	0.0325 (9)	
H8	0.4020	0.0191	0.1321	0.039*	
C9	0.2321 (3)	−0.05851 (11)	0.2449 (7)	0.0422 (10)	
H9	0.2589	−0.0804	0.2644	0.051*	
C10	0.2804 (3)	−0.02736 (10)	0.2493 (7)	0.0376 (10)	
H10	0.3394	−0.0285	0.2688	0.045*	
C11	0.2406 (2)	0.00546 (10)	0.2248 (6)	0.0324 (9)	
C12	0.1522 (3)	0.00546 (10)	0.1922 (7)	0.0399 (10)	
H12	0.1229	0.0269	0.1745	0.048*	
C13	0.1090 (3)	−0.02658 (11)	0.1866 (7)	0.0438 (11)	
H13	0.0502	−0.0265	0.1632	0.053*	

### Atomic displacement parameters (Å^2^)

**Table:**

	*U*^11^	*U*^22^	*U*^33^	*U*^12^	*U*^13^	*U*^23^
N1	0.0402 (19)	0.0294 (16)	0.053 (2)	−0.0098 (14)	0.0033 (17)	−0.0067 (17)
O1	0.057 (2)	0.0575 (15)	0.098 (3)	−0.0119 (15)	0.024 (2)	−0.006 (2)
O2	0.052 (2)	0.0601 (15)	0.097 (4)	0.0054 (14)	0.018 (2)	−0.010 (2)
O3	0.0488 (18)	0.0275 (14)	0.083 (3)	−0.0033 (12)	0.0095 (17)	−0.0067 (17)
O4	0.0399 (16)	0.0334 (15)	0.071 (2)	0.0014 (12)	0.0126 (16)	−0.0045 (15)
C1	0.0301 (19)	0.0442 (18)	0.045 (2)	0.0001 (14)	0.0017 (19)	0.0004 (18)
C2	0.0335 (19)	0.028 (2)	0.046 (2)	0.0031 (15)	−0.0038 (18)	−0.0062 (18)
C3	0.0320 (19)	0.0282 (18)	0.038 (2)	−0.0014 (14)	−0.0008 (18)	0.0009 (17)
C4	0.0294 (19)	0.0310 (18)	0.038 (2)	−0.0053 (14)	0.0011 (16)	0.0001 (17)
C5	0.0300 (19)	0.0292 (18)	0.035 (2)	0.0014 (14)	−0.0026 (17)	−0.0039 (16)
C6	0.0274 (18)	0.0298 (19)	0.037 (2)	−0.0010 (14)	0.0009 (16)	−0.0012 (16)
C7	0.0291 (19)	0.0319 (19)	0.033 (2)	−0.0023 (14)	0.0006 (17)	0.0007 (18)
C8	0.032 (2)	0.0280 (18)	0.037 (2)	0.0021 (15)	0.0010 (18)	−0.0013 (16)
C9	0.048 (2)	0.0270 (19)	0.052 (3)	0.0011 (17)	0.000 (2)	−0.0019 (18)
C10	0.030 (2)	0.0340 (19)	0.049 (3)	0.0004 (15)	0.0029 (19)	−0.0046 (19)
C11	0.0336 (19)	0.028 (2)	0.035 (2)	−0.0015 (15)	0.0038 (17)	−0.0027 (17)
C12	0.032 (2)	0.0314 (19)	0.056 (3)	0.0017 (15)	0.0026 (19)	−0.003 (2)
C13	0.036 (2)	0.038 (2)	0.057 (3)	−0.0054 (17)	−0.002 (2)	−0.005 (2)

### Geometric parameters (Å, °)

**Table:**

N1—C13	1.320 (5)	C5—C6	1.385 (5)
N1—C9	1.329 (5)	C6—C7	1.385 (5)
N1—H1	0.8600	C6—H6	0.9300
O1—C1	1.316 (5)	C7—C8	1.389 (5)
O1—H1A	0.8200	C7—C11	1.484 (5)
O2—C1	1.201 (5)	C8—H8	0.9300
O3—C2	1.233 (5)	C9—C10	1.386 (5)
O4—C2	1.290 (5)	C9—H9	0.9300
C1—C3	1.498 (5)	C10—C11	1.386 (6)
C2—C5	1.502 (5)	C10—H10	0.9300
C3—C4	1.385 (5)	C11—C12	1.397 (6)
C3—C8	1.394 (5)	C12—C13	1.374 (6)
C4—C5	1.379 (5)	C12—H12	0.9300
C4—H4A	0.9300	C13—H13	0.9300
			
C13—N1—C9	120.2 (3)	C6—C7—C8	119.5 (3)
C13—N1—H1	119.9	C6—C7—C11	120.0 (3)
C9—N1—H1	119.9	C8—C7—C11	120.5 (3)
C1—O1—H1A	109.5	C7—C8—C3	119.9 (3)
O2—C1—O1	124.0 (4)	C7—C8—H8	120.0
O2—C1—C3	123.1 (4)	C3—C8—H8	120.0
O1—C1—C3	112.8 (4)	N1—C9—C10	121.0 (4)
O3—C2—O4	124.0 (3)	N1—C9—H9	119.5
O3—C2—C5	120.8 (4)	C10—C9—H9	119.5
O4—C2—C5	115.2 (3)	C11—C10—C9	119.9 (4)
C4—C3—C8	119.7 (3)	C11—C10—H10	120.1
C4—C3—C1	120.9 (3)	C9—C10—H10	120.1
C8—C3—C1	119.3 (3)	C10—C11—C12	117.5 (4)
C5—C4—C3	120.5 (3)	C10—C11—C7	121.8 (3)
C5—C4—H4A	119.7	C12—C11—C7	120.7 (3)
C3—C4—H4A	119.7	C13—C12—C11	119.2 (4)
C4—C5—C6	119.6 (3)	C13—C12—H12	120.4
C4—C5—C2	119.1 (3)	C11—C12—H12	120.4
C6—C5—C2	121.4 (3)	N1—C13—C12	122.2 (4)
C7—C6—C5	120.7 (3)	N1—C13—H13	118.9
C7—C6—H6	119.6	C12—C13—H13	118.9
C5—C6—H6	119.6		
			
O2—C1—C3—C4	−176.6 (5)	C6—C7—C8—C3	−2.0 (6)
O1—C1—C3—C4	−0.2 (6)	C11—C7—C8—C3	178.4 (4)
O2—C1—C3—C8	1.7 (7)	C4—C3—C8—C7	2.2 (6)
O1—C1—C3—C8	178.1 (4)	C1—C3—C8—C7	−176.1 (4)
C8—C3—C4—C5	−0.3 (6)	C13—N1—C9—C10	−0.4 (7)
C1—C3—C4—C5	178.0 (4)	N1—C9—C10—C11	1.5 (7)
C3—C4—C5—C6	−1.9 (6)	C9—C10—C11—C12	−1.3 (7)
C3—C4—C5—C2	179.1 (4)	C9—C10—C11—C7	−179.2 (4)
O3—C2—C5—C4	11.1 (6)	C6—C7—C11—C10	−150.4 (4)
O4—C2—C5—C4	−170.3 (4)	C8—C7—C11—C10	29.3 (6)
O3—C2—C5—C6	−167.9 (4)	C6—C7—C11—C12	31.8 (6)
O4—C2—C5—C6	10.7 (6)	C8—C7—C11—C12	−148.5 (4)
C4—C5—C6—C7	2.2 (6)	C10—C11—C12—C13	0.2 (7)
C2—C5—C6—C7	−178.8 (4)	C7—C11—C12—C13	178.1 (4)
C5—C6—C7—C8	−0.2 (7)	C9—N1—C13—C12	−0.7 (8)
C5—C6—C7—C11	179.4 (4)	C11—C12—C13—N1	0.8 (7)

### Hydrogen-bond geometry (Å, °)

**Table:**

*D*—H···*A*	*D*—H	H···*A*	*D*···*A*	*D*—H···*A*
N1—H1···O4^i^	0.86	1.70	2.562 (4)	175
N1—H1···O3^i^	0.86	2.67	3.252 (4)	126
O1—H1A···O4^ii^	0.82	1.96	2.643 (5)	141
C8—H8···O2^iii^	0.93	2.71	3.632 (5)	171
C10—H10···O2^iii^	0.93	2.58	3.225 (6)	127
C9—H9···O1^iv^	0.93	2.59	3.316 (6)	135

Symmetry codes: (i) −*x*+1/4, *y*−1/4, *z*−1/4; (ii) *x*+1/2, *y*, *z*−1/2; (iii) −*x*+1, −*y*, *z*; (iv) −*x*+3/4, *y*−1/4, *z*+1/4.
